# Novel therapies for myasthenia gravis: Translational research from animal models to clinical application

**DOI:** 10.4103/NRR.NRR-D-24-01011

**Published:** 2025-03-25

**Authors:** Benedetta Sorrenti, Christian Laurini, Luca Bosco, Camilla Mirella Maria Strano, Adele Ratti, Yuri Matteo Falzone, Stefano Carlo Previtali

**Affiliations:** 1 Neurology Unit, IRCCS San Raffaele Scientific Institute, Milan, Italy; 2 Neuromuscular Repair Unit, Institute of Experimental Neurology (INSPE) and Division of Neuroscience, IRCCS San Raffaele Scientific Institute, Milan, Italy; 3 Vita-Salute San Raffaele University, Milan, Italy

**Keywords:** acetylcholine receptor (AChR), animal models, B-cell depletion, biological therapies, complement, immunotherapy, muscle-specific kinase (MuSK), neonatal Fc receptor

## Abstract

Myasthenia gravis is a chronic autoimmune disorder that affects the neuromuscular junction leading to fluctuating skeletal muscle fatigability. The majority of myasthenia gravis patients have detectable antibodies in their serum, targeting acetylcholine receptor, muscle-specific kinase, or related proteins. Current treatment for myasthenia gravis involves symptomatic therapy, immunosuppressive drugs such as corticosteroids, azathioprine, and mycophenolate mofetil, and thymectomy, which is primarily indicated in patients with thymoma or thymic hyperplasia. However, this condition continues to pose significant challenges including an unpredictable and variable disease progression, differing response to individual therapies, and substantial long-term side effects associated with standard treatments (including an increased risk of infections, osteoporosis, and diabetes), underscoring the necessity for a more personalized approach to treatment. Furthermore, about fifteen percent of patients, called “refractory myasthenia gravis patients”, do not respond adequately to standard therapies. In this context, the introduction of molecular therapies has marked a significant advance in myasthenia gravis management. Advances in understanding myasthenia gravis pathogenesis, especially the role of pathogenic antibodies, have driven the development of these biological drugs, which offer more selective, rapid, and safer alternatives to traditional immunosuppressants. This review aims to provide a comprehensive overview of emerging therapeutic strategies targeting specific immune pathways in myasthenia gravis, with a particular focus on preclinical evidence, therapeutic rationale, and clinical translation of B-cell depletion therapies, neonatal Fc receptor inhibitors, and complement inhibitors.

## Introduction

Myasthenia gravis (MG) is a rare chronic autoimmune condition that affects the postsynaptic neuromuscular junction (NMJ). The overall prevalence of MG is estimated to be between 150 and 250 cases per million individuals, with an annual incidence of 8 to 10 cases per million person/year. MG is more common in women under 40 years of age and men over 60, and its incidence appears to increase with age (Gilhus et al., 2019). The predominant clinical manifestation is muscle fatigability, typically fluctuating over time, variably affecting the ocular, bulbar, respiratory, and limb skeletal muscles (Gilhus et al., 2019). Approximately 15% of MG cases remain limited to the ocular muscles, but most patients eventually progress to generalized MG (gMG), involving multiple muscle groups. Most gMG patients have detectable pathogenic antibodies in their serum, with around 85% testing positive for anti-acetylcholine receptor (AChR) antibodies while a smaller percentage has antibodies against muscle-specific kinase (MuSK) or lipoprotein receptor-related protein 4 (LRP4). 10%–15% of patients have no detectable antibodies and are therefore classified as seronegative.

### Animal models of myasthenia gravis

**Figure 1:**
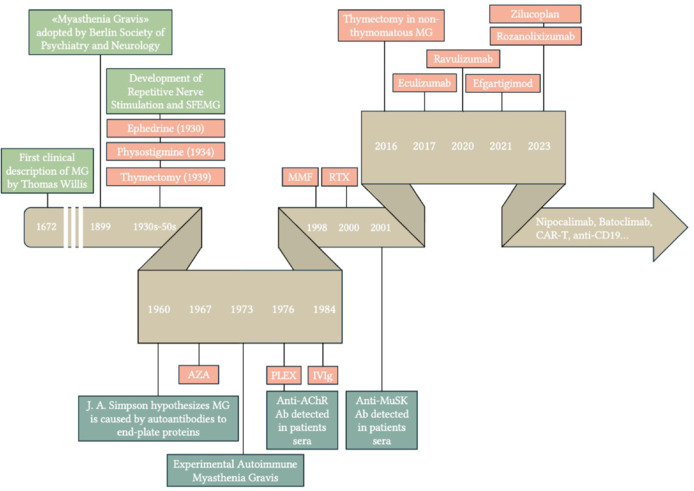
Timeline of key milestones in myasthenia gravis: from discovery to modern therapies. Clinical milestones are highlighted in light green, immunological advancements in dark green, and therapeutic developments in red. The timeline tracks the progression of knowledge on myasthenia gravis from its first clinical description in 1672 through major immunological discoveries and evolving treatment approaches, up to the most recent advancements in therapies. AChR: Acetylcholine receptor; AZA: azathioprine; CAR-T: chimeric antigen receptor T cell; IVIg: intravenous immunoglobulins; MG: myasthenia gravis; MMF: mycophenolate mofetil; MuSK: muscle-specific tyrosine kinase; PLEX: plasma-exchange; RTX: rituximab.

MG was first experimentally induced in rabbits in 1973 (Patrick and Lindstrom, 1973). In their pioneering work, Lindstrom and colleagues demonstrated that immunizing mammals with AChR protein purified from the electric organ of *Electrophorus electricus* triggered an autoimmune response specifically targeting AChRs at the NMJ (Patrick and Lindstrom, 1973; **Figure 1**). This resulted in a condition known as experimental autoimmune myasthenia gravis (EAMG), characterized by the production of anti-AChR antibodies, neuromuscular blockade, and subsequent flaccid paralysis (Patrick and Lindstrom, 1973). The study provided compelling evidence that immunizing rabbits with AChR led to severe paralysis, which could be temporarily alleviated with anticholinesterase drugs like edrophonium or neostigmine. Electromyographic analyses revealed abnormal muscle fatigue, closely mimicking the electrophysiological features of human MG (Patrick and Lindstrom, 1973). Notably, this work established the utility of EAMG as a robust preclinical model to study the pathogenesis of MG and test potential therapies. The disease was subsequently passively transferred using IgG-containing sera derived either from MG patients (Toyka et al., 1975) or from immunized animal models (Lindstrom et al., 1976). Passive transfer myasthenia gravis (PTMG) models enabled the isolation of the humoral response from the broader immune reaction, proving invaluable for investigating the complement-mediated mechanisms of MG pathogenesis, as discussed later in the chapter on complement inhibitors. Following the identification of anti-MuSK autoantibodies, various active and passive models of MuSK-related MG were developed (Richman et al., 2012; Verschuuren et al., 2018). Several of these models successfully replicate key features of MuSK^+^ MG, including muscle atrophy and hypersensitivity to acetylcholinesterase inhibitors.

### Role of autoantibodies in myasthenia gravis

Preclinical evidence showed that antibodies targeting extracellular or transmembrane proteins are causative for the disease, either directly or indirectly, leading to impaired ion transport across the muscle membrane and, consequently, reduced muscle contraction (Kordas et al., 2014; Gilhus et al., 2016).

**Figure 2:**
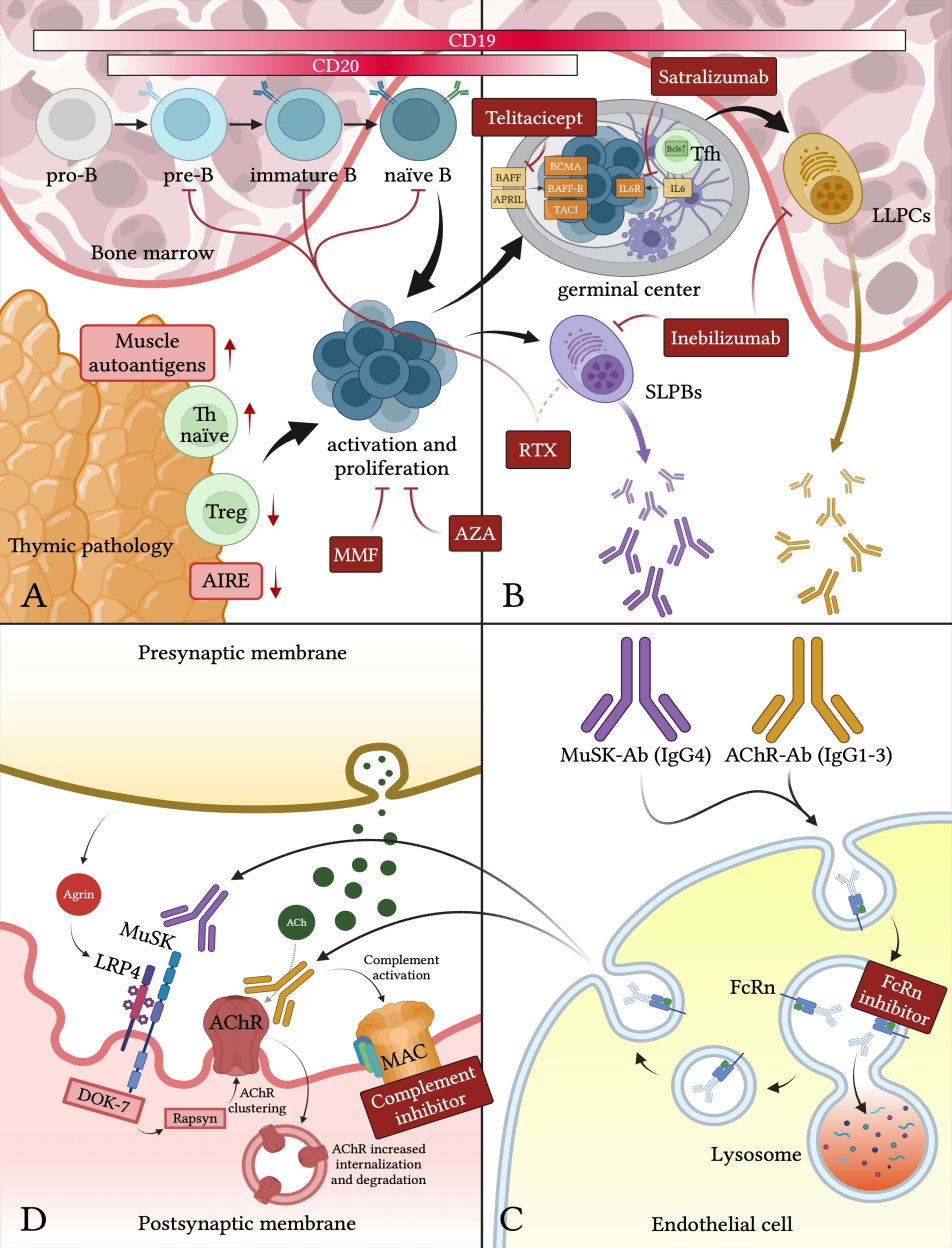
Pathophysiology of myasthenia gravis: from B-cell activation to neuromuscular junction damage. (A) Maturation of B cells and thymic alteration of immune tolerance: The maturation of B cells involves two main stages: an antigen-independent phase that occurs in the bone marrow and an antigen-dependent phase in the lymphoid tissues. Specific CD markers like CD19 and CD20 are useful for identifying the different stages of B cell development. B lymphocytes originate from hematopoietic stem cells in the bone marrow, where they first develop into pro-B, pre-B, immature B, and finally into naïve B cells, independent of antigen exposure. Naïve B cells then recirculate through peripheral lymphoid tissues, where they encounter their specific antigen. This interaction is necessary for activation, proliferation, and terminal differentiation. The thymus is crucial to T cell maturation and the establishment of central tolerance by eliminating self-reactive T cells. This process is facilitated by AIRE, which promotes the expression of various peripheral proteins on the surface of thymic epithelial cells. T cells that recognize these self-antigens are then subjected to negative selection and undergo apoptosis. T cells that evade this central tolerance mechanism are usually managed by regulatory T cells and peripheral tolerance. In Myasthenia gravis, central tolerance fails within the thymus for most patients, leading to the emergence of self-reactive T cells and the formation of autoreactive anti-AChR-antibody-producing B cells. (B) Autoantibodies generation: Based on specific immune processes, plasma cells can be divided into short-lived and long-lived types. Short-lived plasma cells (CD19^+^, CD20^±^) are generated quickly when B cells encounter an antigen, often in secondary lymphoid organs like lymph nodes or spleen. This rapid differentiation typically occurs outside the germinal center in what is known as the extrafollicular response. The local cytokine environment (interleukin-10) during the extrafollicular response often favors the production of IgG4, such as anti-MuSK antibodies. Long-lived plasma cells (CD19^+^, CD20^–^) originate from B cells that undergo a more complex maturation process within the germinal center. During this process, known as affinity maturation, B cells interact closely with follicular helper T cells and undergo somatic hypermutation to enhance the specificity of their antibodies. Only B cells that produce high-affinity antibodies are selected to either become memory B cells or differentiate into long-lived plasma cells which migrate to the bone marrow or other specialized niches where they can survive for months or even years providing sustained antibody production. In Myasthenia gravis, long-lived plasma cells are responsible for the production of anti-AChR antibodies (IgG1–IgG3 subclasses). (C) FcRn mode of action: FcRn is essential for maintaining IgG levels in the bloodstream by preventing their degradation in lysosomes within endothelial cells. IgG enters cells via pinocytosis. Pinocytic vesicles then fuse with acidic endosomes where FcRn binds to IgG at a low pH, preventing their degradation. The FcRn-IgG complex is subsequently transported to the cell surface, where the pH returns to neutral, allowing IgG to be released back into the bloodstream. (D) Pathophysiology of MG at the neuromuscular junction: Anti-AChR antibodies bind the extracellular domain of the receptor impairing signal transduction through three different mechanisms of action: (1) complement activation and formation of the membrane attack complex; (2) antigen modulation leading to increased internalization and degradation of the receptor; (3) blocking the ACh binding site. Anti-MuSK antibodies prevent the interaction between MuSK and LRP4 leading to a reduced AChR clustering on the postsynaptic membrane. Created with BioRender.com. Ab: Antibody; ACh: acetylcholine; AChR: acetylcholine receptor; AIRE: autoimmune regulator; APRIL: A proliferation-inducing ligand; AZA: azathioprine; BAFF: B-cell activating factor; BAFF-R: BAFF receptor; BCMA: B-cell maturation antigen; CD: cluster of differentiation; DOK-7: docking protein 7; FcRn: neonatal fragment crystallizable receptor; Ig: immunoglobulin; LLPCs: long-lived plasma cells; LRP4: lipoprotein receptor-related protein 4; MAC: membrane attack complex; MMF: mycophenolate mofetil; MuSK: muscle-specific kinase; SLPBs: short-lived plasma B cells; TACI: transmembrane activator and CAML interactor; Th: T helper; T reg: T regulator.

Antibodies against nicotinic AChR, which belong to the gamma-globulin class IgG1-3, bind the extracellular domain of the receptor, impairing signal transduction (Kordas et al., 2014; Gilhus et al., 2016). These antibodies operate through three primary pathogenic mechanisms (**Figure 2D**). First, they can activate the complement system, leading to the formation of the membrane attack complex (MAC) and subsequent damage to the postsynaptic membrane; second, they can cause antigenic modulation, where the crosslinking of AChRs by bivalent antibodies accelerates AChR internalization and destruction, resulting in a loss of receptors at the postsynaptic membrane; less commonly, some anti-AChR antibodies directly block the ACh binding site further inhibiting signaling (Kordas et al., 2014; Gilhus et al., 2016).

Recent literature provides new insights into the immunopathology of MG, demonstrating that single autoantibody clones against AChR can efficiently mediate multiple modes of pathology. MG patients consistently produce autoantibodies targeting multiple AChR subunits, reinforcing the idea that multi-subunit specificity plays a key role in triggering the disease (Pham et al., 2023). Competition and inhibition experiments have demonstrated the widespread presence of these multi-specific antibodies across patient groups without any link between antibody specificity and disease severity. Moreover, multiple antibodies can synergize to amplify pathogenic effects, highlighting the complexity of polyclonal AChR-specific autoantibodies in MG (Rose et al., 2022).

Clustering of AChRs at the NMJ is facilitated by MuSK, a transmembrane protein activated through phosphorylation triggered by the LRP4-agrin complex (Huijbers et al., 2014; **Figure 2D**). Anti-MuSK antibodies are found in 1%–10% of MG patients and are predominantly of the IgG4 subclass, which cannot activate complement; additionally, since these antibodies are functionally monovalent, they cannot induce antigen modulation (Huijbers et al., 2013). Instead, anti-MuSK antibodies block the binding sites on MuSK that are necessary for interactions with its binding partners, such as LRP4, effectively inactivating MuSK (Hoch et al., 2001; Huijbers et al., 2013). This inactivation reduces the postsynaptic density of AChRs and disrupts their alignment in the postsynaptic membrane (Hoch et al., 2001). Finally, anti-LRP4 antibodies, mainly of the IgG1 subclass, disrupt the interaction between LRP4 and agrin, indirectly inhibiting MuSK activation and AChR clustering (Higuchi et al., 2011). Additionally, similar to anti-AChR antibodies, they trigger complement activation and lead to IgG deposition at the neuromuscular junction (Higuchi et al., 2011).

These antibodies, regardless of their target, belong to the IgG class and are continuously recycled from intracellular degradation through the neonatal Fc receptor (FcRn) (Stapleton et al., 2015; **Figure 2C**).

The cause of selective muscle autoantibody production in MG is not fully understood. In most AChR^+^ MG patients, the thymus is affected, either through thymoma (10%) or thymic follicular hyperplasia (over 80%) (Marx et al., 2013). Overexpression of muscle autoantigens in thymoma has been linked to the risk of developing MG (Radovich et al., 2018). Thymectomy can improve MG symptoms, suggesting the thymus is central to AChR^+^ MG by disrupting immune tolerance (Wolfe et al., 2016). Furthermore, the development of MG also correlates with the generation of mature naïve T cells in thymomas (Ströbel et al., 2002; **Figure 2A**). However, the role of the thymus in MuSK^+^ MG is less clear, as the organ is without detectable inflammation (Leite et al., 2005).

Advances in immunology, translational medicine, and drug development have led to the emergence of several biological molecules that offer selective, target-specific immunotherapy with fewer side effects and rapid onset of action compared with standard therapies. This review focuses specifically on B-cell targeting agents, FcRn inhibitors, and complement inhibitors due to their promising results in addressing the immune mechanisms underlying MG. By selectively modulating key pathways, these treatments have demonstrated significant potential for improving patient outcomes while minimizing the adverse effects associated with conventional immunosuppressive therapies. Here, we review the development of these new therapies, from preclinical evidence to their implementation in clinical practice (**Figure 1**).

## Search Strategy

To gather relevant literature, we conducted a comprehensive search of the PubMed database using keywords such as “myasthenia gravis,” “autoimmune diseases,” “biologic therapies,” “B-cell depletion,” “FcRn inhibitors,” “complement inhibitors,” and “animal model.” The search was limited to articles published in English up to December 12, 2024. Reviews, clinical trials, and preclinical studies were included if they were relevant to the topics discussed in this review. Reference lists of relevant studies were also examined to ensure comprehensive coverage of the literature.

## B-Cell Targeted Therapy

### Introduction to B cell development and roles

B-cell development begins in progenitor cells (pro-B cells) with the recombination of variable (V), diversity (D), and joining (J) gene segments at the heavy chain locus (VDJ recombination) (LeBien and Tedder, 2008). The resulting μ heavy chain pairs with surrogate light chains to form the pre-B cell receptor (pre-BCR) on pre-B cells (Melchers, 2005). The expression of the pre-BCR is crucial for activating Bruton’s tyrosine kinase and promoting the expansion and survival of the pre-B cells (Rawlings, 1999). The subsequent step involves VJ recombination at the light chain locus, leading to the surface expression of the BCR (basically an IgM) and the production of immature B lymphocytes (Chi et al., 2020). In humans, this mechanism occurs in the adult bone marrow for follicular B cells (LeBien and Tedder, 2008; **Figure 2A**).

This process generates a diverse repertoire of BCRs, which then undergo negative and positive selection (Levine et al., 2000; Nemazee, 2017). B cells that are not autoreactive and persist in the periphery undergo further differentiation, leading to the expression of surface IgM and IgD (Cyster and Allen, 2019). These cells, now termed naïve B cells, recirculate through peripheral lymphoid tissue (lymph nodes and spleen) and wait to encounter their specific antigen (Cyster and Allen, 2019). This leads to activation, proliferation, and differentiation along two main pathways: germinal center B cells undergo somatic hypermutation and produce memory B cells, plasmablasts, and long-lived plasma cells (Cyster and Allen, 2019). Furthermore, a subpopulation of B cells produce short-lived plasma cells outside of the germinal center (Cyster and Allen, 2019; **Figure 2B**). Survival in these intermediate and late stages is promoted by the binding of the B-cell-activating factor of the tumor-necrosis-factor family (BAFF) and A proliferation-inducing ligand (APRIL) to their receptors: B-cell maturation antigen (BCMA), BAFF-receptor (BAFF-R), and transmembrane activator and CAML interactor (Vincent et al., 2013).

T follicular helper (Tfh) cells are crucial in the germinal center, driving B-cell activation, survival, and differentiation into memory cells and plasma cells. The cytokine interleukin-6 (IL-6) facilitates this by promoting the differentiation of naïve T cells into Tfh cells through IL-6 receptor (IL-6R) signaling (Hernández Ruiz et al., 2025). This process induces the expression of Bcl6 (Korn and Hiltensperger, 2021), a transcription factor essential for sustaining Tfh identity and optimizing their interactions with B cells to support germinal center dynamics (Hernández Ruiz et al., 2025).

Beyond their role in antibody production, B cells are involved in the formation of tertiary lymphoid tissues and the secretion of inflammatory cytokines such as interleukin-2, tumor necrosis factor, interferon-γ, IL-6, and granulocyte-macrophage colony-stimulating factor. They also serve as key antigen-presenting cells in the activation of T-cell immunity (Bouaziz et al., 2007; Lee et al., 2021), a role that has been extensively studied in the context of multiple sclerosis (Piccio et al., 2010).

### Preclinical evidence regarding B-cell targeted therapy

B cell depleting therapy is a cornerstone in treating autoimmune diseases driven by humoral immunity such as rheumatoid arthritis and autoimmune encephalitis (Lee et al., 2021). The complexity in this approach lies in understating which cells are the main drivers of antibody production, and how to target them effectively and safely.

Rituximab (RTX) was the first chimeric monoclonal antibody approved for targeting CD20, a B-cell-specific antigen (Pierpont et al., 2018). Initially developed for the treatment of hematological malignancies, its therapeutic applications have since expanded to include several autoimmune diseases (Pierpont et al., 2018). Additionally, RTX is often used off-label in many immune-mediated conditions (Stubgen, 2008), including MG (Tandan et al., 2017; Vesperinas-Castro and Cortés-Vicente, 2023), particularly in cases associated with MuSK antibodies (Vakrakou et al., 2023). Preclinical research on RTX, primarily focused on its oncological applications, has demonstrated multiple mechanisms by which it induces B-cell death (Clynes et al., 2000; Harjunpää et al., 2000; Alas and Bonavida, 2001; Pierpont et al., 2018). These include complement-dependent cytotoxicity, complement-dependent cellular cytotoxicity, antibody-dependent cellular cytotoxicity, antibody-dependent cellular phagocytosis, inhibition of B-cell proliferation, and the induction of apoptosis (Clynes et al., 2000; Harjunpää et al., 2000; Alas and Bonavida, 2001; van Meerten et al., 2006; Pierpont et al., 2018).

CD20 is not a pan-B marker, as it is expressed from the pre-B cell stage but is downregulated during differentiation into antibody-secreting plasma cells (Pierpont et al., 2018). CD19, on the other hand, has a broader expression profile, being expressed from the pro-B stage to most plasma cells (LeBien and Tedder, 2008; **Figure 2A** and **B**). CD19 represents a novel and promising target in B-cell-mediated diseases. Inebilizumab is a humanized anti-CD19 monoclonal antibody that primarily depletes its target cells through antibody-dependent cellular cytotoxicity (Frampton, 2020). This is achieved by engineering its Fc portion to lack a fucose moiety, increasing the antibody’s affinity for FcγRIIIa receptors expressed by macrophages and natural killer cells (Herbst et al., 2010). In humanized CD19/CD20 double transgenic mice, inebilizumab achieved more extensive and longer-lasting bone marrow B-cell depletion compared to RTX, an effect that was independent of complement activation (Herbst et al., 2010). Inebilizumab was recently approved by the US Food and Drug Administration (FDA) and European Medicines Agency (EMA) for anti-aquaporin-4 seropositive Neuromyelitis Optica spectrum disorder (Frampton, 2020) and is being investigated in a phase 3 clinical trial in gMG (NCT04524273).

Other approaches to B-cell depletion are being studied in the context of gMG, although recent trials have yielded mixed results. Preclinical evidence from mouse models of autoimmune diseases has demonstrated that BAFF inhibition is a valuable therapeutic option (Gross et al., 2000, 2001). Belimumab, an anti-BAFF monoclonal antibody, is already approved for treating systemic lupus erythematosus. However, a phase II trial evaluating adjuvant belimumab in patients with gMG did not achieve its primary endpoint (Hewett et al., 2018). Telitacicept represents a more promising alternative and will be discussed later. Furthermore, Bruton’s tyrosine kinase inhibition, a cornerstone in treating various hematological malignancies (Alu et al., 2022), is being studied for a wide range of autoimmune diseases (Lorenzo-Vizcaya et al., 2020; Arneson et al., 2021; Krämer et al., 2023). A phase 3 clinical trial involving a Bruton’s tyrosine kinase inhibitor (tolebrutinib) in gMG (NCT05132569) was recently terminated because of concerns regarding liver toxicity (Sanofi, 2022).

Indirectly modulating B-cell activity by targeting Tfh cells represents a promising therapeutic strategy. Xin et al. (2014) demonstrated that RNA interference targeting Bcl6 effectively mitigated the severity of EAMG, suppressed Tfh cell expansion, and reduced the production of anti-AChR antibodies. More recently, Miyake et al. (2024) reported that treatment of EAMG mice with anti-IL6R antibodies led to enhanced muscle strength, diminished production of anti-AChR antibodies, reduced IgG deposition at the neuromuscular junction, and fewer Tfh cells in lymph nodes. Efforts to target the IL6-IL6R axis in MG patients are discussed in detail later.

### Therapeutic rationale for targeting B cells in myasthenia gravis

Targeting upstream B cells, as opposed to downstream effector pathways, presents obvious theoretical advantages. Evidence has demonstrated the pathogenic role of autoantibodies in MG through various mechanisms (Huijbers et al., 2014). From a theoretical perspective, targeting the B-cell lineage in MG can be effective for three main reasons: (1) direct depletion of cells producing pathogenic antibodies; (2) indirect depletion through the elimination of precursor cells; and (3) antibody-independent effects on cytokine production and regulation of T-cell immunity.

RTX infusion leads to a rapid depletion of circulating B cells, rendering them undetectable within one month, and their numbers remain low for at least 6 months (Dalakas, 2008). CD20^+^/CD27^+^ memory B cells typically begin to reemerge approximately 8 months following RTX administration, and treatment may need to be repeated every 6 to 12 months on average (Dalakas, 2008). Total serum immunoglobulin levels are not significantly affected by RTX infusion (Marco et al., 2014), suggesting they are mainly produced by CD20^–^ long-lived plasma cells. Most antibody-secreting plasma cells are indeed CD20-negative (Pierpont et al., 2018); therefore, RTX would be expected to reduce pathogenic autoantibody levels by decreasing the production of plasma cells rather than by directly eliminating them, an effect that is more pronounced when the half-lives of the autoantibody-producing cells are shorter. Evidence from animal models (Hoyer et al., 2004), other autoimmune conditions (Chihara et al., 2011; Zografou et al., 2021) and isolated case reports (Marino et al., 2020) suggests a particular role of short-lived plasmablasts (SLPBs) in the early autoimmune stages (Wardemann et al., 2003) and in IgG4-mediated diseases (Vakrakou et al., 2023), such as MuSK^+^ MG. Unlike long-lived plasma cells in the bone marrow, SLPBs require constant replenishment from the B-cell compartment (Cyster and Allen, 2019), making B-cell depletion an intriguing strategy in this situation (Lee et al., 2021). Furthermore, a subset of SLPBs expresses CD20 (Zografou et al., 2021). In a mouse model of rheumatoid arthritis, RTX specifically depleted a subset of autoreactive CD20^+^ short-lived, antibody-secreting cells (Huang et al., 2010). However, even during RTX-induced clinical remission in patients with MuSK^+^ gMG, a residual population of CD20^+^ memory B cells may persist despite B cell depleting therapy or reappear over time (Stathopoulos et al., 2017). The expansion of these cells and the subsequent generation of MuSK antibody-producing plasmablasts can predict disease relapses (Stathopoulos et al., 2017; Fichtner et al., 2022). As already stated, targeting CD19 ensures a broader B-cell depletion, including most antibody-secreting cells. Patients treated with inebilizumab showed a significant decrease in total immunoglobulin levels (Rensel et al., 2022), even those who had previously been treated with RTX (Flanagan et al., 2022). Ongoing clinical trials will address concerns related to safety and potential immunosuppression.

Monoclonal antibodies have revolutionized clinical practice but they present some inherent limitations, such as tissue penetration, relatively short half-lives, and suboptimal target-cell killing (Chames et al., 2009). Chimeric antigen receptor (CAR)-T cell therapy exploits engineered T-cell receptors (CARs) to redirect autologous T cells to recognize and eliminate cells expressing a specific antigen (Sterner and Sterner, 2021). Several CAR-T therapies are already approved by FDA and EMA in the context of hematological malignancies (Bellino et al., 2023; Chen et al., 2023), but this platform could have several therapeutic applications in autoimmune diseases as well (Schett et al., 2024). As discussed later, CAR-based approaches targeting CD19, BCMA, or both, are being tested on patients with gMG and have shown promising results (Granit et al., 2023; Haghikia et al., 2023; Oh et al., 2023).

B-cell depletion effectively “resets” the circulating B-cell repertoire, eliminating memory B-cells that replenish antibody-producing plasma cells or may produce pro-inflammatory cytokines (Lee et al., 2021). The reconstituting B cells are enriched in naïve cells with a new and diverse BCR repertoire and in IL-10-producing regulatory B cells (Lee et al., 2021). In addition to antibody production, B-cells play a crucial role in antigen presentation to CD4^+^ T-helper lymphocytes, alongside dendritic cells (Stubgen, 2008; Lee et al., 2021). Activated T-helper cells can then perpetuate chronic inflammation and further B-cell activation in a self-sustaining cycle mediated by IL-17, IL-21, and the BAFF/APRIL pathway (Uzawa et al., 2021).

### Translating B-cell targeted therapy to clinical practice in myasthenia gravis

Since the year 2000 (Zaja et al., 2000), numerous case reports and series have documented the efficacy of RTX in treating refractory gMG (Vesperinas-Castro and Cortés-Vicente, 2023). Several observational studies with relatively small patient samples have been published over the years (Nowak et al., 2011; Collongues et al., 2012; Beecher et al., 2018; Choi et al., 2019; Litchman et al., 2020; Lu et al., 2020; Li et al., 2021), frequently yielding contradictory results. These inconsistencies can be attributed to non-uniform RTX dosing and redosing regimens, differing patient characteristics and primary endpoints, and varying antibody statuses of the patients. At least four different meta-analyses on the use of RTX in MG have been published in recent years (Iorio et al., 2015; Tandan et al., 2017; Feng et al., 2021; Zhao et al., 2021), generally indicating favorable response rates. However, one study reported superior outcomes in MuSK^+^ patients compared to AChR^+^ patients (Tandan et al., 2017).

To date, two placebo-controlled randomized clinical trials on RTX in MG have been completed, while a third is still recruiting patients (NCT05868837). The BeatMG phase II trial included patients with AChR^+^ gMG on a stable dose of prednisone (at least 15 mg/d) (Nowak et al., 2022). Fifty-two patients were randomized to receive either two cycles of RTX (375 mg/m^2^ weekly for four weeks) or matched placebo. The primary endpoint was defined as a ≥ 75% reduction in the mean daily prednisone dose, but the study reached its futility endpoint (60% with RTX *vs.* 56% with placebo), indicating a low probability of achieving a clinically meaningful steroid-sparing effect of 30% due to RTX over placebo in a potential phase III trial with this set of inclusion criteria (Nowak et al., 2022). The RINOMAX study randomized forty-seven patients with onset of gMG in the previous year and a quantitative myasthenia gravis (QMG) score of six or more to receive either a single 500 mg RTX infusion or a matched placebo (Piehl et al., 2022). 71% of treated patients reached the primary endpoint of minimal manifestations (QMG of four or less with prednisolone 10 mg or less daily, and no rescue treatment) at 16 weeks compared to only 29% of patients in the placebo group (Piehl et al., 2022). RTX also reduced the risk of rescue treatment almost ten-fold (4% with RTX *vs.* 36% with placebo; Piehl et al., 2022). These diverging results likely reflect the changing biology of autoantibody production at different time points in the natural history of MG. This hypothesis seems to be confirmed by a retrospective study, in which patients with new-onset MG demonstrated a faster response to RTX compared to refractory cases (Brauner et al., 2020). In the same study, RTX showed a superior safety and efficacy profile compared with conventional immunosuppressant therapies.

In summary, existing evidence indicates that RTX is a safe and generally effective therapy for patients with gMG, with higher efficacy in newly diagnosed patients compared to those with long-standing refractory disease (Brauner et al., 2020; Piehl et al., 2022; Vesperinas-Castro and Cortés-Vicente, 2023). As previously stated, RTX affects most stages of B-cell development but does not directly impact plasma cells (Pierpont et al., 2018). Evidence from other autoimmune diseases suggests that the early stage of autoantibody production is mainly mediated by plasmablasts and SLPBs (Wardemann et al., 2003; Chihara et al., 2011), whereas persistent antigen-driven stimulation triggers the development of RTX-resistant long-lived plasma cells and memory B-cells (Nutt et al., 2015). An increase in the percentage of peripheral blood memory B-cells has been shown to accurately predict a relapse in RTX-treated MG patients (Ruetsch-Chelli et al., 2021).

Regarding MuSK^+^ MG, while there is a lack of data from clinical trials, numerous observational studies have reported that this subclass of patients exhibits a superior (Beecher et al., 2018; Choi et al., 2019; Litchman et al., 2020; Caballero-Ávila et al., 2022; Heckmann, 2022) and more rapid (Stathopoulos et al., 2017; Castiglione et al., 2022) therapeutic response compared to those with AChR^+^ MG. Additionally, clinical remission appears to be more prolonged in the MuSK^+^ subgroup (Díaz-Manera et al., 2012; Topakian et al., 2019; Caballero-Ávila et al., 2022). Investigations into the kinetics of MuSK autoantibodies demonstrate a rapid and sustained decline in these antibodies without significantly affecting total IgG or IgG4 levels, confirming this observed difference (Stathopoulos et al., 2017; Marino et al., 2020; Zhou et al., 2021). Given the well-documented challenges in managing MuSK^+^ MG patients and the inefficacy of complement-targeted therapies, the authors advocate for the consideration of RTX as a first-line therapy in this population (Vakrakou et al., 2023; **Table 1**).

**Table 1:** Molecular therapies for myasthenia gravis in clinical practice

Therapeutic agent	Target	Clinical trials	Patients enrolled	Outcomes (treatment *vs*. placebo)	Dosing regimen	Safety concerns	Clinical indications
**Complement inhibitors**							
Eculizumab	Humanized monoclonal ab anti-C5 Inhibition of terminal complement/MAC activation	REGAIN – phase III randomized placebo-controlled trial	AChR^+^ adult refractory gMG patients, 125 (placebo 63; eculizumab 62)	MG-ADL on week 26: 56.6 *vs*. 68.3; rank-based treatment difference –11.7, 95% CI –24.3 to 0.96, *P* = 0.0698, NS In sensitivity analysis: MG-ADL total score on week 26: –4.2 *vs*.–2.3 (*P* = 0.0058), QMG total score on week 26: –4.6 *vs*. –1.6 (*P* < 0.0006)	900 mg IV weekly for 4 weeks (initial dose), then 1200 mg IV every 2 weeks (maintenance dose)	Increased risk of Neisseria meningitidis infections Mild to moderate headache	Approved by FDA and EMA for the treatment of AChR^+^ refractorya gMG patients.
Ravulizumab	Humanized IgG2/4 monoclonal ab anti-C5 Inhibition of terminal complement/MAC activation	CHAMPION MG – phase III randomized, placebo-controlled trial	AChR^+^ adult gMG patients, 175 (placebo 89; ravulizumab 86)	MG-ADL total score on week 26: –3.1 *vs*. –1.4 (*P* < 0.001). QMG total score on week 26: –2.8 *vs*. –0.8 (*P* < 0.001)	2400–3000 mg IV (initial dose), then 3000–3600 mg IV every 8 weeks (maintenance dose). BW dose regimen (< 60 kg, 60–100 kg, > 60 kg)	Increased risk of Neisseria meningitidis infections	Approved by FDA and EMA for the treatment of AChR^+^ gMG patients.
Zilucoplan	Short 35 kDa macrocyclic peptide anti-C5/C5b Inhibition of terminal complement/MAC activation	RAISE – phase III randomized, placebo-controlled trial	AChR^+^ adult gMG patients, 174 (placebo 86; zilucoplan 89)	MG-ADL total score on week 12: –4.39 *vs*. –2.30 (*P* = 0.0004) QMG total score on week 12: –6.19 *vs*. –3.25 (*P* < 0.0001)	0.3 mg/kg BW daily SC injections	Mild: headache, injection site reactions May increase risk of Neisseria meningitidis infections	Approved by FDA and EMA for the treatment of AChR^+^ gMG patients.
**FcRn inhibitors**							
Efgartigimod	Anti-FcRn-IgG1 Fc fragment Inhibition of autoantibody recycling (IgG3>IgG4)	ADAPT – phase III randomized, placebo-controlled trial	gMG patients, 167 (placebo 84, Efgartigimod 83); AChR^+^ 129; MuSK^+^ 6; seronegative MG 32	Results for total cohort: MG-ADL responders in cycle 1: 68% *vs*. 37%, OR: 3.70 [95% CI: 1.85–7.58] (*P* < 0.001) Results for AChR^+^ patients: MG-ADL responders in cycle 1: 68% *vs*. 30%, OR: 4.95 (95% CI: 2.21–11.53) (*P* < 0.001) QMG responders in cycle 1: 63% *vs*. 14%, OR: 10.84 (95% CI: 4.18–31.20) (*P* < 0.001)	10 mg/kg BW IV once weekly for 4 weeks cycle, repeated as needed based on clinical response no sooner than 8 weeks after initiation of the previous cycle	Mild: headache, nasopharyngitis, reduced monocyte count, injection site reactions	Approved by FDA and EMA for the treatment of AChR^+^ gMG patients. Approved in Japan for the treatment of gMG patients, regardless of their serological status.
Rozanolixizumab	Human anti-FcRn IgG4 ab Reduction of autoantibody levels	MycarinG – phase III randomized, placebo- controlled trial	gMG patients, 200 (placebo 67; rozoanolixizumab 7 mg/kg 66; rozanolixizumab 10 mg/kg 67); AChR^+^ 179; MuSK^+^ 21	Results for total cohort at 7 mg/kg dose: MG-ADL on day 43: –3.37 *vs*. – 0.78 (*P* < 0.0001) QMG on day 43: –5.40 *vs*. –1.92 (*P* < 0.0001) Results for total cohort at 10 mg/kg dose: MG-ADL on day 43: –3.40 *vs*. –0.78 (*P* < 0.0001) QMG on day 43: –6.67 *vs*. –1.92 (*P* < 0.0001)	7 or 10 mg/kg BW SC injections once a week for 6 weeks cycle, repeated as needed based on clinical response	Mild to moderate headache	Approved by FDA and EMA for the treatment of AChR^+^ or MuSK^+^ gMG patients.
ANTI-CD20							
Rituximab	Chimeric monoclonal IgG anti-CD20 Depletion of CD20^+^ cells	BEAT MG – phase II randomized, placebo-controlled trial RINOMAX – randomized, placebo-controlled study	AChR^+^ adult gMG patients, 52 (placebo 27, rituximab 25) AChR^+^ adult gMG patients, 47 (placebo 22, rituximab 25)	Steroid-sparing outcome: 60% *vs*. 56% (futility endpoint *P* = 0.03) Minimal manifestations on week 16: 71% *vs*. 29% (*P* = 0.007)	Cycle regimen every 6 months. Each cycle includes a 375 mg/m^2^ IV weekly infusion for 4 consecutive weeks 500 mg IV single infusion at baseline	Minor risk of infections, infusion site reactions	Used off-label as first line option for the treatment of MuSK^+^ gMG patients.

^a^Refractory MG: Presence of at least one of the following criteria, despite standard treatment (thymectomy if indicated; corticosteroids and at least two other immunosuppressive agents, used at appropriate dosages and for an adequate duration): (1) At least one myasthenic crisis or significant exacerbation event per year with the need for plasmapheresis or immunoglobulins, or (2) need for regular plasmapheresis or intravenous immunoglobulins, or (3) intolerable side effects/comorbidities that limit or contraindicate the use of immunosuppressants. ab: Antibody; AChR: acetylcholine receptor; BW: body weight; CI: confidence interval; EMA: European Medicines Agency; FcRn: neonatal Fc receptor; FDA: US Food and Drug Administration; gMG: generalized Myasthenia gravis; IgG: immunoglobulin G; IV: intravenous; MAC: membrane attack complex; MG-ADL: myasthenia gravis activities of daily life; MuSK: muscle-specific tyrosine kinase; NS: not significant; QMG: quantitative myasthenia gravis; SC: subcutaneous.

Telitacicept is a fusion protein combining a recombinant transmembrane activator and CAML interactor and a human IgG Fc fragment. It functions by sequestering BAFF and APRIL, inhibiting their interaction with receptors and affecting B-cell and plasma cell survival (Dhillon, 2021). Already approved in China for the treatment of active systemic lupus erythematosus, telitacicept is currently under investigation for MG in a phase III trial (NCT05737160) following encouraging reports and positive results from a phase II trial (Lin et al., 2024; Yin et al., 2024).

Recent reports on the use of tocilizumab (an anti-IL6R monoclonal antibody) in MG have emerged, generally showing efficacy (evidenced by reductions in MG-ADL and QMG scores), alongside an acceptable safety profile (Yang et al., 2023; Jia et al., 2024; Ruan et al., 2024). A phase II trial of tocilizumab in MG is currently recruiting patients in China (NCT05067348). Satralizumab, an anti-IL6 monoclonal antibody, was investigated in the LUMINESCE phase III trial (NCT04963270), which demonstrated a favorable safety profile and a significant but modest effect on MG-ADL and QMG scores at week 24 (Habib et al., 2024).

CAR-T cell therapies are being investigated for MG, as exemplified by a clinical case where a 33-year-old woman with refractory gMG was successfully treated with anti-CD19 CAR-T cells (Haghikia et al., 2023). BCMA-targeted CAR-T therapy, which has shown promise in treating multiple myeloma, is also gaining attention for MG, as reported by a case where bispecific BCMA/CD19 CAR-T cells induced remission in a 64-year-old patient (Zhang et al., 2024).

Descartes-08 is a BCMA-targeted CAR-T therapy based on RNA that was studied in a prospective, open-label, phase 1b/2a clinical study (MG-001) (Granit et al., 2023), with promising results and an acceptable safety profile. Another BCMA-targeted CAR-T-based therapy has received approval for a clinical trial for gMG in the US (NCT04561557). Recently, the use of a chimeric autoantibody receptor expressed in T cells has been validated for the specific recognition and targeting of MuSK autoreactive plasma cells *in vitro* (Oh et al., 2023). A consequent phase I trial is currently recruiting patients with MuSK^+^ MG (NCT05451212).

## FcRn Inhibitors

### Introduction to FcRn

FcRn is primarily known for its role in transporting IgG in various tissues, contributing to humoral immunity in newborns and adults, and prolonging the half-life of IgG providing their recycle from intracellular degradation (Stapleton et al., 2015). Structurally, FcRn is an MHC class-I-like molecule composed of three extracellular alpha domains and a trans-membrane domain non covalently bound to β2-microglobulin, which is essential to obtain a functional protein (Simister and Mostov, 1989; Zhu et al., 2002; Stapleton et al., 2015). It is most highly expressed in hematopoietic cells, intestinal epithelia, and the vascular endothelium but it mediates also many other functions across a variety of tissues, such as the kidney, mammal gland, lung, liver, and brain (Kuo et al., 2010).

The existence of FcRn as a receptor involved in the transfer of maternal IgG from mother to infant was first suggested in the 1960s (Brambell, 1966) and, lately, confirmed by other groups providing biochemical evidence of IgG transport from the gut epithelial cells of neonatal rats (Rodewald, 1976). Subsequently, FcRn has been studied for its critical role in maintaining serum levels of both IgG and albumin by rescuing these molecules from lysosomal degradation (Junghans and Anderson, 1996) through a pH-dependent mechanism mediated by histidine residues in the C_H_2-C_H_3 hinge region of IgG (Vaughn and Bjorkman, 1998; Oganesyan et al., 2014). FcRn binds IgG in acidic endosomes, preventing its degradation and returning it to the plasma membrane for release into the bloodstream as the pH normalizes. This process extends the half-life of IgG compared to other immunoglobulins (**Figure 2C**). Albumin serum levels are regulated through the same mechanism, although IgG and albumin bind to FcRn at distinct, non-cooperative sites (Chaudhury et al., 2003, 2006).

Recently, the involvement of FcRn has been characterized in various other mechanisms, including the bidirectional transport of IgG and albumin across polarized cellular barriers (Spiekermann et al., 2002; Yoshida et al., 2006), as well as its involvement in enhancing responses to IgG-immune complexes (Hubbard et al., 2020), phagocytosis (Zhu et al., 2001; Vidarsson et al., 2006) and antigen presentation (Baker et al., 2014). FcRn is pivotal for effective antigen presentation, enhancing immune responses, and facilitating mucosal vaccination. The interaction between IgG and FcRn influences the intracellular fate of immune complexes, contributing to the processing of the antigen into peptide epitopes that can bind to both MHC class I and class II molecules. This dual activation process of CD4^+^ and CD8^+^ T cell responses effectively links the humoral and cellular branches of the adaptive immune system and is essential in contexts such as tumor immunity and responses to various pathogens (Baker et al., 2013, 2014; Macri et al., 2021).

### Preclinical evidence of therapeutic applications of IgG-FcRn interactions

The FcRn function in regulating the transport and recycling of IgG can be employed by antibody engineering as a therapeutical approach to modulate the levels of either treatment-related or endogenous IgG. Mouse models provided the first evidence that FcRn deficiency leads to an accelerated clearance of endogenous antibodies (Ghetie et al., 1996; Israel et al., 1996; Liu et al., 1997), while FcRn overexpression results in increased serum IgG concentrations (Lu et al., 2007; Cervenak et al., 2011). Based on these observations and advances in FcRn knowledge, research has focused on using antibody engineering to modulate FcRn-IgG interactions. By the late 1990s, it was first demonstrated that specific mutations in the Fc region of mouse IgG affecting its binding to mouse FcRn could either shorten or lengthen their serum half-live (Kim et al., 1994; Ober et al., 2001). Later, investigations have focused on human IgG antibodies engineered to enhance acid-dependent binding to human FcRn in primate and rodent models (Hinton et al., 2004; Vaccaro et al., 2005). Altogether, these findings reveal that altering Fc regions to enhance their binding affinity to human FcRn prolongs the presence of therapeutic antibodies in the serum.

A similar approach can be employed in treating autoimmune diseases to remove endogenous pathogenic IgG. Evidence shows that direct inhibition of FcRn and its interaction with IgG promotes the breakdown of pathogenic antibodies, similar to how intravenous immunoglobulins (IVIg) act in treating autoimmune conditions. Preclinical research confirms that IVIg shortens the half-life of pathogenic antibodies by competitively binding to FcRn (Lünemann et al., 2015).

Similarly, engineered antibodies known as antibodies that promote the degradation of IgG (AbDegs) have been demonstrated to outcompete native IgG, leading to accelerated catabolism (Petkova et al., 2006). AbDegs are designed to bind FcRn with a higher affinity at both acidic and neutral pH levels and enter cells mainly by receptor-mediated processes, competing very effectively with endogenous IgGs for FcRn interactions (Vaccaro et al., 2005).

The proof-of-principle that decreasing IgG levels by targeting FcRn could be therapeutically relevant for treating autoimmune diseases comes from disease models, such as rat EAMG and mouse experimental arthritis, where AbDegs reduce total IgG levels by approximately 60% (Liu et al., 2007a; Patel et al., 2011). Mice and non-human primate models showed a reduction in all IgG subclasses, including IgG4, without significant changes in serum levels of albumin and other immunoglobulin (IgA, IgM) when FcRn is antagonized (Nixon et al., 2015). Moreover, FcRn antagonism did not impair the generation of primary and secondary immune responses.

More recently, it has been proven that the interaction between FcRn and IgG can also be modulated using small molecules or peptide inhibitors. One such small molecule, named SYN1436, was administered at repeated doses of 5 mg/kg three times per week in cynomolgus monkeys, leading to an approximately 80% reduction in IgG levels while leaving albumin concentrations unchanged (Mezo et al., 2008).

### Therapeutic rationale for targeting FcRn in myasthenia gravis

Given the antibody-mediated pathogenesis of MG and the effectiveness of antibody-depleting therapies such as IVIg and plasma exchange, particularly during disease exacerbations, FcRn has emerged as an attractive therapeutic target for MG (Guptill et al., 2016).

Preclinical research using experimental animal models of MG supports this treatment strategy. The role of a high affinity, pH-independent anti-rat FcRn inhibitor, named 1G3, has been investigated in both passive and active EAMG demonstrating significant dose-dependent reduction of IgG levels and disease symptoms (Huijbers et al., 2019). Furthermore, the therapeutic potential of FcRn inhibitors was also proven in a mouse model of MuSK^+^ MG, as confirmation that this category of drugs leads to a reduction in the levels of all IgG subclasses, including IgG4 (Liu et al., 2007b).

In light of these encouraging results, numerous molecules have been investigated as possible treatments for MG. Among FcRn peptide inhibitors, the most promising agent was efgartigimod, a hIgG1-derived Fc fragment designed using AbDeg technology to enhance its affinity for FcRn at both neutral and acid pH, as shown by *in vitro* studies with transfected endothelial cells (Ulrichts et al., 2018). In cynomolgus monkeys, doses up to 20 mg/kg of efgartigimod reduced IgG levels by 75% without significant safety issues. Similar findings were confirmed also in humans, with the 10 mg/kg dose showing the greatest IgG reduction and being selected for further clinical development (Ulrichts et al., 2018).

Similar results have been reached also with various monoclonal antibodies directed to FcRn. The first evidence about the efficacy of a humanized high-affinity anti-human FcRn monoclonal antibody, named IgG4P (rozanolixizumab), in exhibiting similar binding affinity to both human and cynomolgus monkey FcRn at pH 6.0 and pH 7.4, comes from a set of *in vitro* studies with various anti-FcRn antibody formats confirming IgG4P being notably more effective than others in blocking IgG recycling (Smith et al., 2018). In the same years, *in vivo* studies with transgenic FcRn mice and cynomolgus monkeys showed dose-dependent reductions in plasma IgG levels, ranging from 75% to 90% below baseline, without significantly impacting plasma albumin concentration (Smith et al., 2018).

Meanwhile, additional preclinical evidence has supported the efficacy of RVT-1401/HBM9161 (batoclimab), a fully human monoclonal IgG1 antibody that effectively reduces IgG serum levels in both animal models and humans, offering valuable insights to future clinical research in gMG patients (Collins et al., 2019; Yap et al., 2021).

Similarly, M281, another fully human monoclonal IgG1 antibody (Ling et al., 2019), and SYNT001, a humanized, de-immunized IgG4 monoclonal antibody with a S241P mutation (Blumberg et al., 2019), were well-tolerated in non-human primates and led to a dose-dependent decrease in serum IgG.

Beyond their consistent effectiveness in reducing serum IgG levels, these molecules have also shown comparable safety profiles. No significant effects were observed on other immunoglobulin classes or complement levels, indicating a notable safety advantage over techniques such as plasma exchange, which removes all serum proteins and can lead to broader immunosuppression and a higher risk of infections (Kiessling et al., 2017). Monoclonal antibodies targeting FcRn seem to generally cause a transient, dose-dependent reduction in serum albumin levels, which resolves after the drug is withdrawn. Moreover, since albumin regulates cholesterol transport and facilitates the movement of cholesterol between plasma lipoproteins, red blood cells, and liver cells, a decrease in circulation albumin may lead to a dose-related increase in blood lipid levels which were reversible after stopping a treatment or might be prevented by co-administration of statins, as noted in subsequent clinical studies (Kahaly et al., 2023).

In contrast, small molecules tend to have a lesser impact on albumin and cholesterol levels. Their specific steric configuration prevents them from interfering with the albumin binding site, resulting in minimal effects on these parameters.

### Translating FcRn inhibition to clinical practice in myasthenia gravis

#### Efgartigimod

Based on promising preclinical evidence, in 2021, a landmark phase III randomized, placebo-controlled trial (ADAPT) demonstrating the efficacy of efgartigimod for the treatment of AChR^+^ gMG was published (Howard et al., 2021a). Key eligibility criteria included an MG-ADL score of at least five and a stable dose of at least one immunosuppressive therapy. A total of 216 patients (129 AChR^+^, 38 AChR^–^, including 6 MuSK^+^) were enrolled. A higher proportion of AChR^+^ patients in the efgartigimod group were MG-ADL responders in cycle 1 compared to placebo (*P* < 0.0001), with similar results for QMG responders. In AChR- patients, response rates were similar, but efgartigimod showed more responders and patients with minimal symptom expression, despite these results not being statistically significant due to the small sample size. Significant reductions in IgG and AChR antibodies were observed, with no decrease in albumin. Adverse events were mostly mild to moderate.

Further data supporting the therapeutic role of efgartigimod in gMG patients come from the open-label extension study, ADAPT^+^, which aimed to assess the long-term safety, tolerability, and efficacy of efgartigimod administered in cycles of four intravenous infusions with a personalized cycle repetition schedule based on clinical needs (Howard et al., 2024). Significant MG-ADL and QMG score improvements were observed as early as one week in both AChR^+^ and AChR^–^ groups, correlating with IgG and anti-AChR antibody reduction.

Given the substantial clinical improvements obtained in clinical trials, efgartigimod was initially approved for treating AChR^+^ gMG in adults in the USA in 2021 and, immediately after, in Europe in 2022. The treatment has also been assessed in other countries, receiving approval in Japan in January 2022 for gMG patients regardless of their antibody status (Heo, 2022). Currently, it is administered at a scheduled dose of 10 mg/kg in cycles of four weekly intravenous doses, repeated depending on clinical response (**Table 1**).

Since its initial approval, early real-world evidence on the use of efgartigimod in clinical practice has reinforced its efficacy. Italian centers reported experiences with nineteen and thirty-one gMG patients of various serological statuses, followed for at least fourteen months under the expanded access protocol (Frangiamore et al., 2024; Pane et al., 2024). Significant improvements in MG scores were noted after each treatment cycle for both AChR^+^ and triple-seronegative patients, though data for MuSK^+^ and LRP4^+^ patients were inconsistent due to small sample sizes. These findings, along with other individual reports, suggest the potential role of efgartigimod for broader use, especially in seronegative patients, highlighting the need for further studies to explore its efficacy in these groups (Frangiamore et al., 2023; Sorrenti et al., 2024).

Similarly, the Israeli (Fuchs et al., 2024) and Japanese (Konno et al., 2024) groups, involving twenty-two and four AChR^+^ gMG patients respectively, showed that efgartigimod significantly reduced MG scores and prednisone doses. Two patterns were observed: in Pattern A, the clinical condition improved after each treatment cycle but worsened and required another cycle after several weeks. In Pattern B, the clinical condition improved after the first cycle and stabilized, but residual disability remained, which improved after another treatment cycle. These data suggest that efgartigimod can offer a flexible administration schedule through its cyclic use, allowing for a customized therapy based on the patient’s clinical needs.

Finally, experiences such as those reported by a Chinese group, which included fifty-six cases of gMG treated with efgartigimod mainly in cases of acute MG exacerbation and myasthenic crisis, highlight the potential role of efgartigimod in treating the acute phase of the disease due to its rapid action (Luo et al., 2024).

#### Rozanolixizumab

More recently, rozanolixizumab has been also approved as add-on subcutaneous treatment in both AChR^+^ and MuSK^+^ gMG. The first trial evaluating its safety, pharmacokinetics, and pharmacodynamics in humans, with different administration routes, subcutaneous and intravenous, revealed that the intravenous form resulted in a dose-dependent increase in adverse events (Kiessling et al., 2017). The highest single doses of rozanolixizumab reduced mean maximum IgG levels by approximately 48% for the intravenous form and 43% for the subcutaneous form, with reductions in serum IgG persisting for weeks. Consequently, clinical development has focused on the more tolerable subcutaneous formulation used in the phase III randomized, placebo-controlled trial MycarinG, which enrolled a total of two hundred gMG patients, both AChR^+^ or MuSK^+^ (Bril et al., 2023). Rozanolixizumab demonstrated consistent and clinically significant efficacy in patients with gMG with over a quarter achieving minimal symptom expression. Both the 7 and 10 mg/kg doses were effective, with the 10 mg/kg dose showing greater improvements in several secondary endpoints. Notably, twenty-one MuSK^+^ gMG patients had more pronounced score reductions than the overall population. Rozanolixizumab caused rapid reductions in IgG concentrations by day eight, correlating with efficacy improvements. Common adverse events, such as headaches and infections, were similar between groups but slightly higher in the 10 mg/kg group. Albumin levels remained stable.

This trial led to the first approval of rozanolixizumab in the USA in June 2023 as an add-on treatment to standard therapy for both groups of patients, AChR^+^ and MuSK^+^ gMG (Hoy, 2023). A few months later, in January 2024, it was approved in Europe with the same indications (**Table 1**). In light of the recent approval, real-life usage data are not yet available; however, open-label trials (NCT05681715) and expanded access programs are currently underway (Bril et al., 2023).

#### Batoclimab

Alongside FcRn inhibitors that have already been approved for clinical use, there are several molecules currently under investigation for the treatment of gMG with promising results.

In healthy volunteers, batoclimab was found to be well-tolerated and safe with both single (intravenous or subcutaneous) and multiple (subcutaneous) doses leading to a dose-dependent reduction in serum IgG of up to approximately 70%, without any severe safety concern (Collins et al., 2019; Yap et al., 2021). Consequently, it was first studied in AChR^+^ gMG patients in a phase II clinical trial, which demonstrated that a low-volume subcutaneous injection of batoclimab, 340 mg or 680 mg, was well-tolerated and significantly reduced total IgG and anti-AChR antibody levels (Yan et al., 2022; Nowak et al., 2024).

The confirmation that batoclimab increases the rate of sustained MG-ADL improvement, as defined by a three-point or greater reduction in the score, in gMG patients comes from a clinical trial conducted by a Chinese research group (Yan et al., 2024). In the trial, one hundred seventy-eight patients, including one hundred twenty-four who were AChR^+^ and five who were MuSK^+^, were assigned to receive batoclimab or placebo with standard care. Batoclimab, given as six weekly 680 mg subcutaneous injections, led to significant MG-ADL improvements in 58.2% of patients, with 4.7% achieving minimal symptoms. Rapid efficacy was noted in MG-ADL and QMG scores by weeks two and one, respectively, and was sustained. Batoclimab also reduced serum IgG and AChR antibody levels. Total cholesterol and LDL-C levels increased in the batoclimab group, peaking at week six but returning close to baseline levels afterward. Similarly, plasma albumin levels decreased initially but rebounded after treatment.

Further evidence of the efficacy of batoclimab in treating gMG will be provided by the phase III FLEX trial as soon as the results become available (Benatar et al., 2024). This pivotal study, designed to assess the efficacy and safety of batoclimab in gMG by evaluating batoclimab doses of 680 and 340 mg once weekly versus placebo, uses an induction-maintenance strategy to offer rapid and sustained efficacy with the flexibility to adjust doses based on patient needs.

#### Nipocalimab

Nipocalimab is also being investigated as a potential treatment for MG, following promising evidence of its safety and effectiveness in reducing IgG levels in healthy volunteers (Ling et al., 2019). Currently, the only data available on the efficacy and tolerability of nipocalimab in patients with gMG come from the vivacity phase II trial (Antozzi et al., 2024). This dose-ranging study evaluated the safety and efficacy of nipocalimab in patients with gMG who did not respond adequately to standard therapy. As a result of the statistical analysis, nipocalimab was found to be generally safe and well-tolerated, with no severe adverse events. A positive dose response in the MG-ADL total score was observed, with the greatest reduction seen in the 60 mg/kg every 2 weeks group. A rapid reduction in total serum IgG, up to 83%, and AChR autoantibodies was noticed.

The incidence of infections was low and similar to the placebo group. Modest, dose-dependent, and reversible increases in cholesterol levels were observed but the overall impact on cardiovascular risk was minimal due to concomitant increases in HDL cholesterol.

The study demonstrated dose-dependent reductions in MG-ADL scores and supported the initiation of a phase III confirmatory study for nipocalimab in gMG (NCT04951622). Although the results are not yet published, the company recently announced positive outcomes at the European Academy of Neurology (EAN) 2024 Congress. In this double-blind, placebo-controlled study, patients receiving nipocalimab plus standard of care showed superior improvement in MG-ADL score over twenty-four weeks compared to those receiving placebo plus standard of care. Nipocalimab treatment resulted in a 4.70-point improvement in MG-ADL scores, significantly more than the 3.25-point improvement observed with placebo (*P* = 0.002). The study included a broad population of AChR^+^, MuSK^+^, and LRP4^+^ patients, representing about 95% of the gMG population. Additionally, safety and tolerability were consistent with previous studies, with similar incidences of adverse events.

## Complement Inhibitors

### Introduction to the complement system

The complement system is a crucial component of the innate immune response, playing a key role in pathogen elimination and immune regulation (Warwick et al., 2021). It consists of a series of proteins that, upon activation, contribute to inflammation, opsonization, and the formation of the MAC. In autoimmune diseases, dysregulation or overactivation of the complement system can exacerbate tissue damage and contribute to disease pathogenesis (West et al., 2024).

AChR antibodies initiate the classical complement pathway, one of the three routes of complement activation, alongside the alternative and lectin pathways (Warwick et al., 2021). This process begins with C1q binding to the Fc region of anti-AChR antibodies, triggering the complement cascade. Key events include the cleavage of complement component C3 into C3a and C3b, and C5 into C5a and C5b. C5b then recruits additional complement components (C6, C7, C8, and C9) to form the MAC (C5b-9) (Lee and Woodruff, 2021). The MAC induces focal lysis of the postsynaptic membrane, disruption of the postsynaptic folds, and ultimately a decrease in functional AChRs (Lindstrom, 1980; **Figure 2D**). The role of the complement system in MG has been extensively demonstrated through both patient studies and animal models. Over the years, preclinical evidence has elucidated the disease’s pathogenesis and guided the development of targeted therapies. These insights have led to the clinical translation of complement-targeting drugs for AChR^+^ MG forms.

### Preclinical evidence of complement’s role in the pathogenesis of myasthenia gravis

The role of complement as a pivotal player in the pathogenesis of MG has been postulated since the 1950s, based on the pioneering studies by Nastuk and Osserman. A key observation supporting this hypothesis was that serum from MG patients, when applied to frog muscle fibers, induced significant changes in muscle electrophysiology and caused cytolytic damage to the fibers, suggesting a potential role for complement-mediated mechanisms (Nastuk et al., 1959, 1960).

During the acute phase of EAMG, the neuromuscular endplates undergo significant macrophagic invasion. This process is driven by chemotactic fragments released from activated complement and is further facilitated by the binding of antibodies and complement components to the postsynaptic membrane (Lennon et al., 1976, 1978; Lindstrom, 1980). The role of complement as a chemotactic agent was further supported by evidence showing that phagocytic invasion was prevented when the rats were pre-emptively depleted of the complement component C3 (Lennon et al., 1976, 1978). Macrophage-mediated attacks play a crucial role in the destruction of the NMJ by inducing alterations in the postsynaptic membrane, which lead to functional denervation of muscle fibers. Additionally, this enhances the effects of antibodies bound to AChR during the acute phase of EAMG (Lindstrom, 1980).

The role of complement extends beyond its chemotactic function, as demonstrated in the chronic phase of EAMG, which more accurately reflects the disease process in humans. In chronic EAMG, the postsynaptic membrane shows fewer and smaller folds, and macrophages are absent. During this phase, antibodies bind to the postsynaptic membrane, causing localized damage. This complement-mediated destruction, following the binding of anti-AChR antibodies, disrupts neuromuscular transmission and directly contributes to the loss of AChRs. Additionally, complement-mediated mechanisms likely play a role in damaging the NMJ folded structure (Engel et al., 1976; Sahashi et al., 1980).

In 1980, Sahashi et al. confirmed the deposition of IgG and C3 in the NMJ of a patient with MG through histological analysis, reflecting the histological findings depicted in EAMG. Furthermore, immunohistochemical and electron microscopy studies conducted on intercostal muscles of patients with gMG undergoing thymoma surgery demonstrated the presence of IgG, C3, and C9 deposits at the NMJ endplates. However, IgG and C3 deposits were predominantly observed in anatomically intact regions, whereas C9, the terminal effector of the complement cascade, is primarily present in severely anatomically altered end plate regions at the NMJ. This distribution provides unequivocal evidence of the antibody-mediated, complement-dependent mechanisms underlying the alteration of NMJ morphology in MG (Sahashi et al., 1980).

Further evidence supporting the role of complement in the pathogenesis of the disease comes from in vitro studies by Appel. These studies demonstrated that 50% of serum from patients with generalized MG AChR^+^ induced cellular lysis and AChR degradation in cultured rat myotubes when incubated with complement (Ashizawa and Appel, 1985). In the absence of complement, incubation of cultures with serum from patients with gMG led to a reduction in the number of AChRs on the myotubes, while AChR degradation was observed. The AChR reduction was attributed to complement-independent mechanisms, suggesting that other factors contribute to the pathology of MG. These findings underscore the potential heterogeneity in the disease mechanisms underlying MG, indicating that while complement plays a significant role, additional, complement-independent processes may also drive the disease.

Research involving mice with deficiencies in specific upstream components of the complement system has provided direct evidence of the complement classic pathway’s involvement in EAMG pathology. Findings indicate that mice deficient in C3 or C4 show a markedly reduced frequency of active EAMG compared to wild-type mice. This reduction is accompanied by lower levels of IgG, C3, and MAC deposition at the NMJ (Tüzün et al., 2003). In contrast to the findings for complement components C3 and C4, research involving genetic deletion of the lectin pathway component revealed no significant difference in susceptibility to EAMG following AChR immunization. Mice lacking this component exhibited comparable levels of IgG, C3, and MAC deposition at the neuromuscular junction, indicating that the lectin pathway does not play a critical role in the development of EAMG (Li et al., 2009). Beyond the upstream components of the complement system, research has demonstrated a crucial role for terminal complement components in the pathology of EAMG. Mice deficient in C5 or C6 exhibited outcomes analogous to those observed with upstream component deficiencies. Specifically, C5-deficient mice displayed a significantly lower incidence of active EAMG, while C6-deficient mice showed resistance to passive EAMG induction. Notably, the administration of human C6 to C6-deficient mice during EAMG induction restored disease pathology to levels comparable to wild-type mice, underscoring the essential role of the MAC in mediating neuromuscular junction damage (Kusner and Kaminski, 2012; Lee and Woodruff, 2021).

These findings underscored the crucial role of complement in the pathogenesis of MG, highlighting its potential as a therapeutic target and paving the way for novel treatment strategies.

### Therapeutic rationale for targeting complement in AChR^+^ myasthenia gravis

The earliest preclinical therapeutic approach dates to 1989, when Biesecker demonstrated that passive PTMG development in mice could be inhibited by using an antibody targeted against C6 (Biesecker and Gomez, 1989). Inhibition of C6 prevented muscle weakness, electrophysiological abnormalities, and loss of AChRs associated with acute EAMG (Lindstrom, 1980). C3 deposition was observed at the NMJ, but macrophages and MAC were not detected. Despite these insights, the authors focused on the pathophysiological role of complement without exploring therapeutic implications. In 2007, an additional approach involving enzymatic inhibition targeting complement regulatory proteins was applied in rats to prevent the development of EAMG. This strategy aimed to modulate the complement system more precisely by interfering with the regulatory proteins that control complement activation. The use of these inhibitors sought to reduce the pathological effects of complement activation and thereby mitigate the onset and progression of EAMG (Hepburn et al., 2008). In the same year, drawing on evidence from other complement-mediated diseases, such as paroxysmal nocturnal hemoglobinuria, an anti-C5 monoclonal antibody (eculizumab) was tested in rats to prevent PTMG. The authors demonstrated that the anti-C5 monoclonal antibody (eculizumab) was effective in inhibiting the development of EAMG in rats compared to untreated controls. It also improved symptoms in rats once EAMG was induced and reduced MAC deposition at the NMJ. Ultrastructural analysis of NMJs in diaphragms from untreated rats revealed simplification of junctional folds, widened synaptic clefts, and electron-dense material indicative of disruption of the synaptic membrane structure. In contrast, NMJs from diaphragms of rats treated with the anti-C5 mAb exhibited only minimal changes, highlighting the therapeutic potential of targeting complement in EAMG (Zhou et al., 2007). Another C5 inhibitor, rEV576, a recombinant protein derived from tick saliva, was evaluated in both PTMG and EAMG models. This agent proved to be highly effective in moderating disease severity in both models, even when administered after the onset of pronounced muscle weakness (Soltys et al., 2009). Similar findings were documented for anti-C1q Ab, which significantly reduced the clinical severity of EAMG (Tüzün et al., 2007).

These preclinical findings led to the translation of these therapies into human clinical trials. Recently, the same therapeutic targets have been addressed using advanced technologies such as small interfering RNA (siRNA). ALN-CC5, a subcutaneously administered N-acetylgalactosamine-conjugated siRNA, effectively silences C5 expression in the liver, leading to significant and sustained suppression of serum C5 levels in wild-type rodents. In non-human primates, this treatment achieved up to 97.5% reduction in serum C5 and corresponding inhibition of complement activity. ALN-CC5 demonstrated efficacy in improving disease symptoms in two standard rat models of MG. Notably, intermediate levels of complement inhibition were sufficient for therapeutic benefit, suggesting that complete elimination of complement activity may not be necessary.

Additional data supporting the therapeutic rationale for targeting complement, though less prominent due to diverging results, come from studies investigating complement activation in patients with MG. Initial research found fluctuations in complement activity correlated with disease severity (Nastuk et al., 1960). More recent studies have reported inconsistent associations between complement components and clinical measures. However, some studies observed decreased levels of complement components like C2 and C5, while others found increased activation products such as C3b and C5a. Although AChR^+^ MG patients tend to show higher complement consumption and activation compared to healthy controls or MuSK^+^ MG, the variations in findings suggest considerable individual differences and the need for further investigation (Aguirre et al., 2020; Iacomino et al., 2022; Stascheit et al., 2023).

### Translating complement inhibition to clinical practice in AChR^+^ myasthenia gravis

In 2017, a landmark Phase III randomized placebo-controlled trial, REGAIN, evaluating the therapeutical efficacy and safety of eculizumab for AChR^+^ adult refractory gMG was published (Howard et al., 2017). Eculizumab, as previously mentioned, is a fully humanized monoclonal antibody that binds with high affinity to C5, inhibiting its cleavage and preventing MAC formation (Howard et al., 2017). Eculizumab was administered intravenously, starting with a 900 mg dose on day 1 and weeks 1, 2, and 3, followed by 1200 mg at week 4, and 1200 mg every two weeks for maintenance. The study cohort included 125 patients with MGFA classes II-IV, with an MG-ADL scores higher than six and refractory gMG, characterized by inadequate response to at least two lines of immunosuppressive treatments. The primary efficacy endpoint was the change in the total MG-ADL score from baseline to week 26. The worst-rank analysis, used as a statistical test in the trial, failed to show a significant difference between eculizumab and placebo due to the overall lack of substantial improvement in the primary and secondary endpoint measures. However, prespecified sensitivity analyses revealed positive results, showing that eculizumab led to improvements in MG-ADL and QMG scores by weeks 1 and 2, with most benefits becoming evident by week 12 and sustained through week 26. The most frequently reported adverse events included headaches, upper respiratory tract infections, and nasopharyngitis. Most of these events were mild to moderate in severity and were not linked to the study drug (Howard et al., 2017). Considering the results from the REGAIN trial, and particularly the effectiveness demonstrated in the sensitivity analysis on a difficult-to-treat cohort like the refractory gMG group, the FDA and EMA approved eculizumab in 2017 (**Table 1**). The innovative aspect of eculizumab, particularly when compared to the immunosuppressive therapies available at the time, lies in its rapid onset of action. This is evidenced by data from the open-label extension and further highlighted by case reports demonstrating its effectiveness in refractory myasthenic crisis (Muppidi et al., 2019; Howard et al., 2021b; Strano et al., 2022; Vinciguerra et al., 2023). Additionally, a further strength of eculizumab became evident during the open-label extension, where the daily dose of background immunosuppressive therapy was reduced in 47% of patients, and 16.2% of patients were able to discontinue immunosuppressive therapy entirely, suggesting a steroid-sparing effect (Dhillon, 2018). Another advantage of eculizumab is its safety profile, which is supported by years of scientific research in other autoimmune conditions along with promising preliminary efficacy data in pediatric forms of gMG (Nishimura et al., 2023; Brandsema et al., 2024).

In 2022, the Phase III CHAMPION study, a randomized, double-blind, placebo-controlled trial, was published, evaluating the efficacy of ravulizumab in adult anti-AChR^+^ gMG (Vu et al., 2022). Ravulizumab, a humanized monoclonal antibody targeting C5, is engineered with an extended half-life, allowing for less frequent dosing (administered intravenously every 8 weeks with the dosage adjusted according to body weight) compared to eculizumab, following an initial loading phase (Vu et al., 2022). The study cohort consisted of 175 patients, with the primary endpoint and inclusion criteria mirroring those of the REGAIN trial; however, the study did not specifically target refractory gMG. The trial successfully met its primary endpoint, with improvements in MG-ADL and QMG scores observed as early as 1 week after starting ravulizumab treatment and sustained throughout the 26-week treatment period (Vu et al., 2022). The proportion of patients experiencing adverse events, including those deemed related to the trial agent by investigators, was similar between the ravulizumab and placebo groups. Considering this evidence, and with the added benefit of extended dosing intervals compared to eculizumab, ravulizumab was approved by the FDA and EMA in 2022 (**Table 1**). Given its similar efficacy, risk profile, and rapid onset of action, ravulizumab is replacing eculizumab in clinical practice.

In 2023, the RAISE trial, a randomized, double-blind, placebo-controlled Phase 3 study, evaluated the efficacy of zilucoplan in adult gMG AChR^+^ patients. Zilucoplan is a small 15-amino acid macrocyclic peptide that, similarly to the previously mentioned drugs, targets C5 to inhibit its activation (Howard et al., 2023). However, unlike eculizumab and ravulizumab, zilucoplan sterically blocks the binding of C5b to C6, thereby preventing the assembly and activity of the membrane attack complex (Howard et al., 2023). The drug was administered subcutaneously at a dose of 0.3 mg/kg each week. The study cohort, consisting of 174 patients, had characteristics comparable to those in the CHAMPION trial, with the primary endpoint focused on the difference in MG-ADL scores at 12 weeks. Zilucoplan successfully met its primary endpoint, showing a significant improvement in the MG-ADL score at 12 weeks. Additionally, it achieved all secondary endpoints, demonstrating comprehensive efficacy across multiple measures of disease severity by the 12-week period (Howard et al., 2023). Moreover, the responder rate was significantly higher in the zilucoplan-treated group compared with the placebo group, with 73% of patients achieving at least a 2-point reduction in MG-ADL and 58% achieving at least a 3-point reduction in QMG. Although not statistically significant, only 5% of patients on zilucoplan required rescue therapy, compared to 12% in the placebo group. Furthermore, 14% of patients on zilucoplan reached minimal manifestation status, compared to 6% in the placebo group. No significant safety concerns emerged compared to placebo, with the primary side effect being localized reactions at the injection site.

Cemdisiran and pozelimab form a promising combination therapy for AChR^+^ gMG. Pozelimab is a fully human IgG4 monoclonal antibody against C5, while cemdisiran is a siRNA targeting mRNA for C5, reducing its hepatic synthesis and circulating levels. Recent studies in non-human primates demonstrated that this combination more effectively inhibits complement activity, providing more durable and complete suppression compared with monotherapy with either drug alone. This has led to ongoing clinical trials investigating the efficacy of combining intravenous pozelimab and subcutaneous cemdisiran in gMG (Cavalcante et al., 2024).

In conclusion, over the past eight years, C5 inhibitors have revolutionized the therapeutic management of AChR^+^ gMG patients. Their efficacy, rapid onset of action, lack of pharmacological interference, and favorable safety profile make them preferable to traditional steroids and immunosuppressive therapies. Ravulizumab and zilucoplan differ in their administration methods—ravulizumab is administered intravenously every eight weeks, while zilucoplan is administered subcutaneously every week—each with its advantages and disadvantages. Currently, no direct comparative studies between these drugs exist; only meta-analyses are available. Therefore, definitive conclusions regarding their relative efficacy cannot be drawn, and dedicated comparative studies are needed.

## Conclusions

Preclinical evidence gathered over the decades, starting from the pioneering studies of the 1950s, has laid the foundation for understanding the pathogenic mechanisms underlying MG. In addition to the understanding of pathogenesis, a further crucial step has been the improvement and advancement of biotechnologies, which have enabled the development of targeted therapies that have led to the current therapeutic landscape, a perfect example of the success of translational research. Modern medicine aspires to provide patients with a precise and, above all, individualized therapeutic approach (Schork, 2015). Personalizing therapy in MG is crucial, given that patients are characterized by clinical heterogeneity, variability in therapeutic response, and diverse antibody profiles, which likely reflect different pathogenic mechanisms in MG patients (Ma et al., 2024). Therefore, a further qualitative step in the therapeutic landscape would be to identify predictors of response to innovative biological therapies, allowing for a more personalized treatment approach.

The currently available biological therapies for MG consist of three main approaches: targeting autoreactive B cells, blocking the FcRn recycling system, and inhibiting complement function. Although B-cell targeting is the least developed therapeutic approach at present, it offers an advantage over the other strategies by potentially achieving a disease-modifying effect, as it acts upstream in the pathogenic process to eradicate autoreactive clones that produce autoantibodies. RTX is an effective therapy for MuSK^+^ MG, but its results are more variable in AChR^+^ forms (Iorio et al., 2015). This variability may be due to autoreactive B cells that sustain the autoimmune response against the NMJ changing over time, potentially losing CD20 expression and giving rise to resistant long-lived plasma cells and memory B cells (Nutt et al., 2015). Therefore, as highlighted by the RINOMAX trial, early use of RTX may lead to better outcomes (Piehl et al., 2022). The use of inebilizumab, targeting CD19, could potentially overcome the limitations of RTX. While this therapy is promising due to its broader depletion of the B-cell lineage, its safety profile still needs to be clearly established (Frampton, 2020). Another approach, CAR T-cell therapy, also presents certain limitations. These include the risk of severe adverse effects, such as cytokine release syndrome and neurotoxicity (Sterner and Sterner, 2021).

FcRn inhibitors have demonstrated solid efficacy in the treatment of AChR^+^ MG and show potential applicability in treating MuSK^+^ MG and seronegative forms (Zhu et al., 2023). However, only rozanolixizumab is approved for MuSK^+^ MG, and efgartigimod is indicated for seronegative forms exclusively in Japan (Heo, 2022; Hoy, 2023). An additional advantage of this therapy is its potential for personalization, as alternative dosing regimens beyond those currently used could prove effective (Howard et al., 2024). The logic for using FcRn inhibitors is particularly strong in patients who have responded to previous treatments with IVIg or PLEX, regardless of their antibody profile. No significant safety concerns have emerged, and there are no requirements for specific vaccinations or notable drug interactions (Li et al., 2024). However, studies focusing on the late-onset MG population, which is increasingly relevant from an epidemiological perspective, would be essential to evaluate whether there is a heightened susceptibility to infections and thus reconsider the vaccination plan. There are currently no data on their use during pregnancy, and there is a possibility that they may interfere with the transfer of IgG antibodies to the fetus (Patel and Bussel, 2020). Most of these drugs are administered subcutaneously, and efgartigimod is expected to be available in this formulation in the future, addressing the issues of cyclicity and the inconvenience of periodic visits to the treatment center.

Anti-complement therapy is exclusively indicated for AChR^+^ MG due to the involvement of complement activation, which does not occur in MuSK^+^ forms (Vakrakou et al., 2023). There are currently no data on its efficacy in LRP4^+^ and seronegative forms. Unfortunately, there are no established predictors of response to this type of therapy, making it unclear which subtypes of AChR^+^ patients should be considered ideal candidates. The safety profile has been well-defined through a long post-marketing history, with the main concern being an increased risk of Neisseria meningitidis infection; however, mandatory vaccination has kept the incidence of such events extremely rare (Muppidi et al., 2019). A previous study has suggested the potential safety of the drug during pregnancy, making it a viable option for young AChR^+^ MG patients (Sarno et al., 2019). Ravulizumab, due to its engineered structure, offers the advantage of extended dosing intervals thanks to its prolonged half-life. Zilucoplan, furthermore, provides the benefit of subcutaneous administration but requires daily dosing.

In conclusion, the flourishing therapeutic landscape offers clinicians a variety of treatment approaches. However, there remains a need for better strategies to guide patients toward specific therapies, and revisiting the study of disease pathogenesis and the role of antibodies in preclinical models could help identify biomarkers for better patient stratification. Moreover, there are no direct comparative efficacy data, only findings from meta-analyses, which limit the generalizability of these results. A significant challenge is the extremely high cost of these treatments, which currently makes them unsustainable on a global scale. Addressing the economic burden of these therapies is essential to ensure broader accessibility and long-term viability within healthcare systems worldwide.

## Data Availability

*Not applicable*.
